# Prevalence and quantification of viable *Campylobacter* species in retail chicken: a comparative study using viability digital polymerase chain reaction and conventional culture methods

**DOI:** 10.3389/fmicb.2026.1995295

**Published:** 2026-10-20

**Authors:** Ayaka Okada, Aimi Fuyuki, Michihiro Shimizu, Md. Jannat Hossain, Yasuo Inoshima

**Affiliations:** 1 Laboratory of Food and Environmental Hygiene, Joint Department of Veterinary Medicine, Faculty of Applied Biological Sciences, Gifu University, Gifu, Japan; 2 Education and Research Center for Food Animal Health, Gifu University (GeFAH), Gifu, Japan; 3 Joint Graduate School of Veterinary Sciences, Gifu University, Gifu, Japan; 4 Laboratory of Veterinary Anatomy, Joint Department of Veterinary Medicine, Faculty of Applied Biological Sciences, Gifu University, Gifu, Japan; 5 Department of Microbiology and Public Health, Faculty of Veterinary, Animal and Biomedical Sciences, Khulna Agricultural University, Khulna, Bangladesh

**Keywords:** *Campylobacter*, culture method, digital PCR, propidium monoazide, viable but non-culturable

## Abstract

*Campylobacter* is a major foodborne pathogen and a significant public health concern, frequently associated with the consumption of undercooked poultry. Although conventional microbiological culture remains the gold standard for detection, it often fails to detect bacteria in the viable but non-culturable (VBNC) state, potentially underestimating the risk to consumers. To address this limitation, we conducted a year-long epidemiological investigation of retail chicken using propidium monoazide-digital polymerase chain reaction (PMA-dPCR). Between February 2025 and January 2026, 120 retail chicken samples were collected from three local supermarkets (10 samples per month). Comparative analysis of PMA-dPCR and conventional culture methods revealed that PMA-dPCR without enhancer yielded a higher overall detection rate (55.0%) than direct culture (43.3%) and enrichment culture (46.7%), whereas PMA-dPCR with enhancer showed a lower detection rate (33.3%). Specifically, the detection of PMA-dPCR-positive/culture-negative samples during multiple months of the study period indicated the presence of membrane-intact *Campylobacter* populations that may include cells in the VBNC state in the retail poultry supply chain. Although lower detection rates were observed in early spring, PMA-dPCR enabled the detection of membrane-intact *Campylobacter* populations that were not detected by conventional culture. These results highlight the value of advanced molecular tools such as PMA-dPCR for a more comprehensive assessment of *Campylobacter* contamination.

## 1 Introduction

*Campylobacter* species, particularly *C. jejuni* and *C. coli*, remain the primary bacterial agents responsible for foodborne gastroenteritis worldwide (World Health Organization, 2020). In Japan, *Campylobacter* food poisoning remains the most frequently reported bacterial foodborne illness in terms of the number of outbreaks, with 220 outbreaks and 1,226 cases reported in 2025 (Ministry of Health, Labour and Welfare, 2026). Recent surveillance studies have also demonstrated frequent *Campylobacter* contamination of retail poultry meat in Japan. Furukawa et al. (2017) detected *C. jejuni* and *C. coli* in 64 and 14% of retail poultry meat samples, respectively, while Sasaki et al. (2023) isolated *Campylobacter* spp. from 39.5% of retail chicken products and reported geographical and seasonal variations in contamination. These findings indicate that retail poultry meat represents an important potential source of human exposure to *Campylobacter* in Japan. Although regulatory surveillance of *Campylobacter* spp. has traditionally relied on culture-based enumeration methods (Commission Regulation (EU) 2017/1495, 2017), these methods may underestimate the number of viable cells because they cannot detect cells in the viable but non-culturable state (VBNC). Therefore, they may be insufficient when used alone for comprehensive risk assessment (Stingl et al., 2021). Under environmental stressors common in the food supply chain—such as refrigeration or atmospheric oxygen exposure—*Campylobacter* spp. can transition into a VBNC state (Yagi et al., 2022). These cells maintain metabolic activity and pathogenic potential despite their inability to form colonies on standard agar media (Baffone et al., 2006), leading to an underestimation of the actual bacterial load in retail poultry meat (Okada et al., 2023).

Culture-independent molecular detection methods, such as polymerase chain reaction (PCR)-based assays, provide a rapid alternative to culture-based detection of *Campylobacter* spp. However, these methods detect DNA regardless of cell viability and may amplify DNA derived from dead cells, resulting in an overestimation of viable bacterial contamination. To overcome this limitation, propidium monoazide (PMA) treatment has been integrated with molecular assays to selectively amplify DNA from viable cells (Nocker et al., 2006). Our previous study successfully optimized a PMA-quantitative PCR (PMA-qPCR) protocol to discriminate membrane-intact from membrane-compromised *Campylobacter* populations (Okada et al., 2022). However, the PMA enhancer reagent increases membrane permeability, thereby facilitating PMA penetration into membrane-compromised and VBNC cells and further excluding their signals (Hossain et al., 2025). Therefore, comparing PMA treatment with and without the enhancer may provide further insight into the physiological status of *Campylobacter* populations present in retail poultry meat.

Digital PCR (dPCR) partitions a reaction mixture into numerous individual microreactions and enables absolute quantification of target DNA based on the number of positive partitions, without the need for standard curves (Hindson et al., 2011). This feature makes dPCR particularly useful for quantifying low levels of bacterial contamination in complex food matrices, where PCR inhibitors and low target concentrations may affect qPCR-based quantification. Because *Campylobacter* contamination of chicken meat is generally present at relatively low levels (Dhillon et al., 2006), dPCR is considered suitable for its detection and quantification. We therefore previously optimized a PMA-digital PCR (PMA-dPCR) method for detecting low-level membrane-intact *Campylobacter* contamination in chicken meat samples (Hossain et al., 2025). This approach may enable more accurate assessment of the risks associated with membrane-intact *Campylobacter* contamination in retail chicken meat.

In this study, we conducted a 12-month longitudinal survey of retail chicken meat and compared culture-based methods, dPCR without PMA treatment, PMA-dPCR with enhancer, and PMA-dPCR without enhancer. We aimed to evaluate the prevalence and estimated quantity of membrane-intact *Campylobacter* populations throughout the year and characterize the differences in membrane integrity among populations in poultry meat. This approach enabled quantitative characterization of membrane-intact *Campylobacter* populations and evaluation of temporal variations in their detection and culturability in the retail environment.

## 2 Materials and methods

### 2.1 Chicken meat and sample treatment

A total of 120 chicken meat packs (whole legs) were purchased from three local supermarkets in Gifu Prefecture, Japan, between February 2025 and January 2026. Each month, three samples were purchased from each of two supermarkets and four samples from the third supermarket, maintaining the same sampling distribution throughout the study period. Samples were transported to the laboratory in isothermal boxes, and isolation of *Campylobacter* spp. was initiated within 2 h of purchase.

Chicken samples weighing 125 g were rinsed with 40 mL of phosphate-buffered saline (PBS) in a stomacher bag. The rinse solution was centrifuged at 8,000 × *g* for 10 min at 4 °C to precipitate *Campylobacter* spp. The resulting pellet was resuspended in approximately 1 mL of PBS and the suspension was divided into five 200-μL aliquots for parallel analyses:

(1) Direct agar plate counting for the enumeration of culturable cells.
(2) Enrichment culture for qualitative detection of *Campylobacter* spp.
(3) DNA extraction without PMA to quantify total bacterial DNA (from both viable and dead cells) via dPCR.
(4) PMA-dPCR without enhancer to estimate membrane-intact bacterial populations by reducing signals derived from membrane-compromised cells.
(5) PMA-dPCR with enhancer to further suppress signals from membrane-compromised cells and provide a more stringent estimate of membrane-intact bacterial populations, as the enhancer is known to suppress signals from membrane-compromised cells, and has previously been shown to reduce the detection of VBNC *Campylobacter* cells (Hossain et al., 2025).

This experimental design enabled a comprehensive comparison of culturability and membrane integrity-based viability estimates and allowed the detection of culture-negative, membrane-intact populations that may include cells in the VBNC state.

### 2.2 Culture-based enumeration

For direct enumeration, 200 μL of the sample suspension (equivalent to 25 g of the original chicken meat) was divided into two 100-μL aliquots, each of which was spread onto modified charcoal-cefoperazone-deoxycholate agar (mCCDA; Oxoid, Basingstoke, Hampshire, U. K.). The plates were incubated at 42 °C for 48 h under microaerobic conditions using an anaerobic jar (Mitsubishi Gas Chemical, Tokyo, Japan) to determine the number of culturable cells (CFU/25 g).

Additionally, *Campylobacter* spp. were detected according to the NIHSJ-02 guidelines (the standard method in Japan) (Momose et al., 2013) with slight modifications. Instead of solid meat samples, a 200-μL aliquot of the sample suspension (equivalent to 25 g of the original chicken meat) was inoculated into 10 mL of Preston selective broth (nutrient broth No. 2 base; Oxoid) supplemented with 5% sterile lysed horse blood, *Campylobacter* Growth Supplement (Oxoid), and Modified Preston *Campylobacter* Selective Supplement (Condalab, Madrid, Spain). Following enrichment at 42 °C for 24 h under microaerobic conditions, cultures were streaked onto mCCDA and incubated at 42 °C for an additional 48 h. Presumptive colonies were isolated and confirmed as *Campylobacter* spp. by colony PCR using the C412F and C1228R primers (Linton et al., 1996).

### 2.3 PMA treatment with or without PMA enhancer

PMA treatment was performed as described previously (Okada et al., 2022). Briefly, 50 μL of either PBS or PMA enhancer for Gram-negative bacteria (5 × solution; Biotium, Inc., Fremont, CA, United States) was added to each 200-μL sample. Subsequently, PMAxx solution (20 mM in H_2_O; Biotium, Inc.) was added to the 250-μL mixtures to achieve a final concentration of 50 μM.

The samples were incubated in the dark at 37 °C for 10 min, followed by light exposure for 15 min using an LED Crosslinker 30 (Takara, Kusatsu, Japan) to activate PMAxx. For the second PMA treatment, the samples were centrifuged at 8,000 × *g* for 10 min at 4 °C, and the resulting pellets were resuspended in 200 μL of PBS. After adding another 50 μL of PBS or PMA enhancer, the samples were treated with 50 μM PMAxx as described earlier.

Finally, the samples were centrifuged at 8,000 × *g* for 10 min at 4 °C, resuspended in 900 μL of PBS to remove residual PMAxx, and centrifuged again. The final pellets were stored at −30 °C until DNA extraction.

### 2.4 DNA extraction and dPCR

DNA was extracted from both PMA-treated and untreated samples. For untreated samples, 200 μL aliquots were centrifuged at 8,000 × *g* for 10 min at 4 °C, and the resulting pellets were stored at −30 °C. Total genomic DNA was extracted using a DNeasy Blood & Tissue Kit (Qiagen, Hilden, Germany) according to the manufacturer’s instructions, with an elution volume of 100 μL.

dPCR analysis was performed using a QIAcuity One, 2plex system (Qiagen). To quantify *Campylobacter* spp., specific primers (campF2-595 and campR2) and a probe (camp2probe) targeting the 16S rRNA gene were utilized (Hossain et al., 2025). The dPCR mixture contained 4 μL of template DNA and was prepared to a final volume of 40 μL using the 4 × Probe PCR Master Mix (QIAcuity Probe PCR Kit, Qiagen) according to the manufacturer’s instructions.

Nuclease-free water was used as a non-template control. QIAcuity Nanoplate 26 k 24-well plates (Qiagen) were employed for absolute quantification. Thermal cycling conditions were as follows: 95 °C for 2 min, followed by 50 cycles of 95 °C for 15 s and 60 °C for 1 min. Data were analyzed using a one-dimensional scatterplot. The resulting concentrations (copies/μL) were converted to DNA copies per 25 g of chicken meat. Because major foodborne *Campylobacter* species, such as *C. jejuni* and *C. coli*, typically contain three copies of the 16S rRNA gene (Taylor et al., 1992), the total copy number was divided by three to estimate cell equivalents. A sample was considered positive when at least one positive partition was detected. A non-template control was included in each dPCR run, and the run was considered valid only when no positive partitions were detected in the non-template control. Because formal LoB and LoD were not established, low-level positive results were interpreted cautiously considering potential analytical variation.

### 2.5 Statistical analysis

Statistical analyses were performed using EZR (Kanda, 2013). Differences in estimated *Campylobacter* spp. counts among the three molecular methods—dPCR without PMA, PMA-dPCR without enhancer, and PMA-dPCR with enhancer—were evaluated using the Friedman test. When a significant difference was detected, pairwise comparisons were performed using the Wilcoxon signed-rank test with Bonferroni correction. Differences in detection rates among the three molecular methods were analyzed using McNemar’s test with Bonferroni correction. Paired detection results between PMA-dPCR without enhancer and each culture method were additionally compared using McNemar’s exact test, and concordant and discordant results were summarized in contingency tables. All tests were two-tailed, and *p* < 0.05 was considered statistically significant.

## 3 Results

### 3.1 Comparison of detection methods in retail chicken meat samples

A total of 120 retail chicken meat samples were collected monthly from February 2025 to January 2026 and analyzed using culture-based and molecular detection methods. Direct culture detected *Campylobacter* spp. in 52 of 120 samples (43.3%), whereas Preston enrichment culture detected *Campylobacter* spp. in 56 of 120 samples (46.7%). In comparison, dPCR without PMA showed the highest positivity rate, detecting *Campylobacter* spp. DNA in 87 of 120 samples (72.5%). PMA-dPCR without enhancer yielded positive results in 66 of 120 samples (55.0%), whereas PMA-dPCR with enhancer detected it in 40 of 120 samples (33.3%).

Monthly detection patterns differed across the detection methods (Figure 1). dPCR without PMA showed higher positivity rates than culture-based methods throughout most of the study period. Among the three molecular methods, PMA treatment showed significantly reduced positivity rates compared with dPCR without PMA (McNemar’s test with Bonferroni correction, *p* = 0.007). The addition of PMA enhancer further reduced positivity rates compared with PMA-dPCR (*p* < 0.001). Higher positivity rates were observed during the summer months, particularly from July to September, across both culture-based and molecular assays.

**Figure 1:**
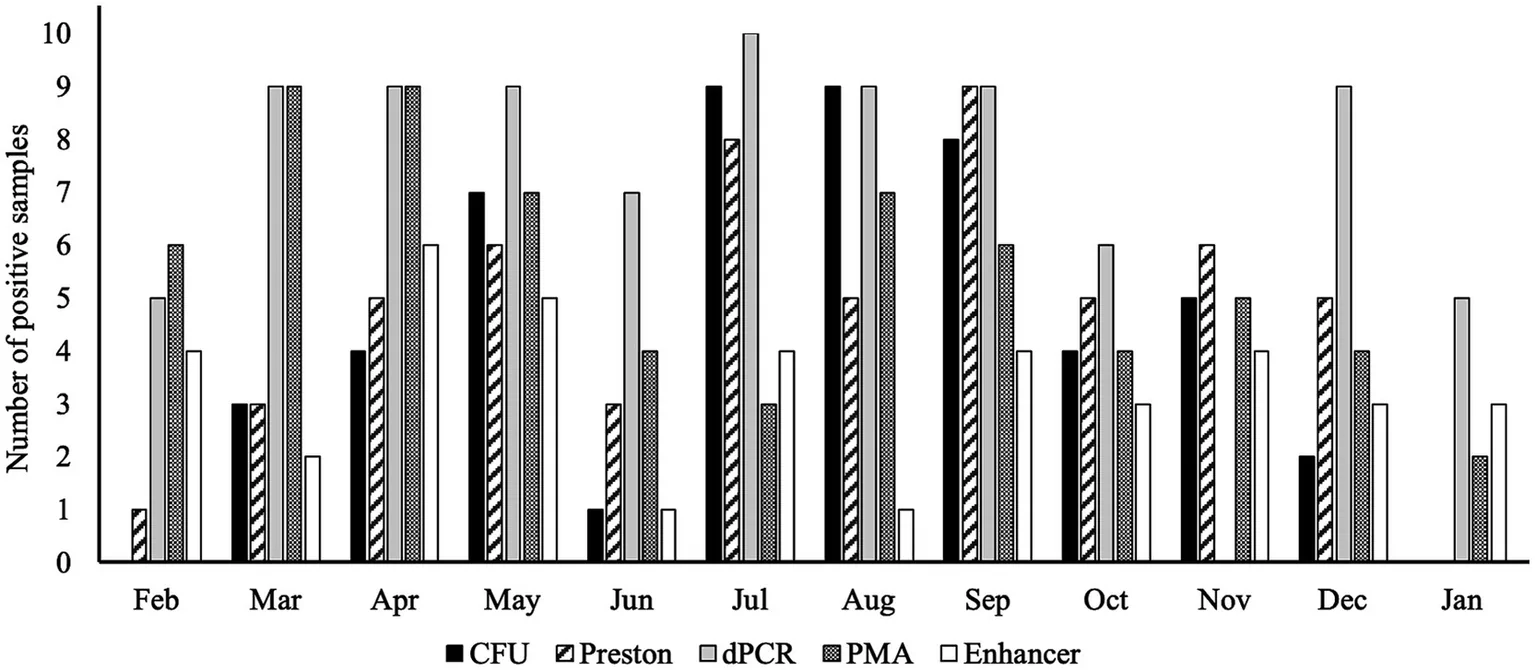
Monthly detection of *Campylobacter* in retail chicken meat by direct plating (CFU), Preston enrichment culture (Preston), dPCR without PMA treatment (dPCR), PMA-dPCR without enhancer (PMA), and PMA-dPCR with enhancer (Enhancer). Ten retail chicken samples (whole legs) were analyzed each month from February 2025 to January 2026. Bars represent the number of positive samples detected by each method. dPCR, digital PCR; PMA, propidium monoazide.

Paired comparisons between PMA-dPCR without enhancer and the culture methods showed that 31 samples were positive by PMA-dPCR but negative by direct culture, whereas 17 samples showed the opposite pattern. Similarly, 27 samples were positive by PMA-dPCR but negative by Preston enrichment culture, whereas 17 samples showed the opposite pattern. The differences in paired detection rates were not statistically significant for either direct culture (McNemar’s exact test, *p* = 0.059) or Preston enrichment culture (*p* = 0.174) (Supplementary Table S1).

### 3.2 Comparison of estimated *Campylobacter* spp. cell equivalents among molecular methods

Quantitative dPCR data were further analyzed to compare the three molecular approaches. Representative samples are shown in Figure 2; for each month, the two samples with the highest dPCR counts were selected for visualization only, whereas statistical comparisons were performed using the complete dataset of all 120 samples. Gene copy numbers were converted to estimated cell equivalents by assuming three copies of the 16S rRNA gene per *Campylobacter* cell.

**Figure 2:**
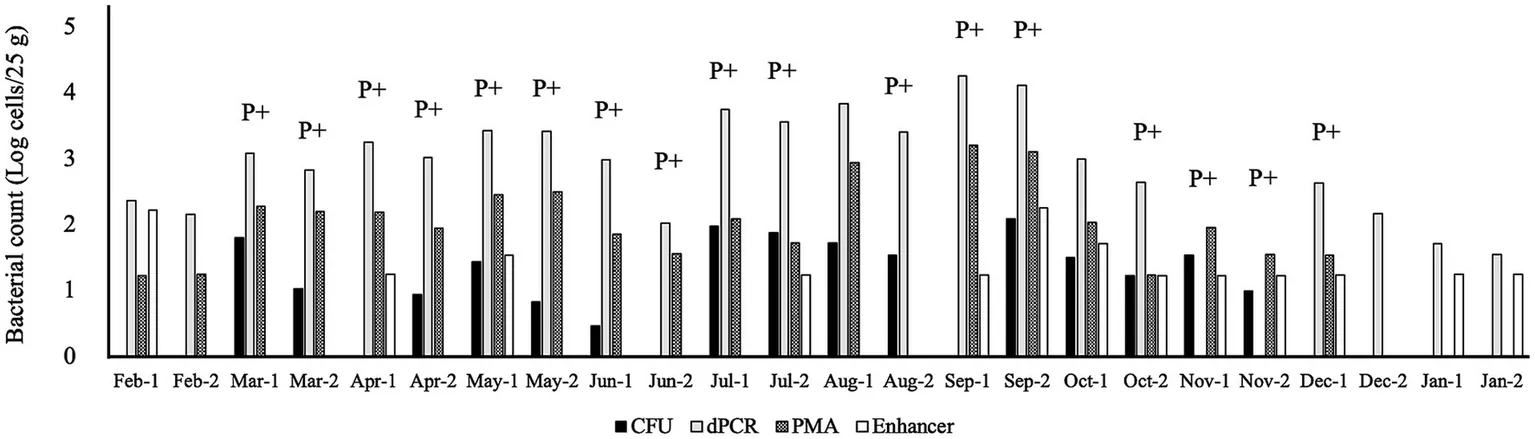
Estimated *Campylobacter* spp. cell equivalents in representative retail chicken meat samples determined by direct plating (CFU), dPCR without PMA (dPCR), PMA-dPCR without enhancer (PMA), and PMA-dPCR with enhancer (Enhancer). Two samples with the highest dPCR counts were selected from each month (February 2025 to January 2026). These samples were selected for visualization only, whereas statistical comparisons among the three molecular methods were performed using the complete dataset of all 120 samples. Gene copy numbers were converted to estimated cell equivalents by assuming three copies of the 16S rRNA gene per *Campylobacter* cell, and the resulting values are expressed as estimated cell equivalents per 25 g of chicken meat. P + above each bar indicates samples positive by Preston enrichment culture. dPCR, digital PCR; PMA, propidium monoazide.

In the representative samples, dPCR without PMA generally showed higher estimated *Campylobacter* spp. counts than PMA-treated assays. PMA treatment reduced detectable counts in many samples, and the addition of PMA enhancer resulted in further reductions or complete loss of detectable signal in several samples.

Statistical analysis of all 120 samples showed significant differences in estimated cell equivalents across the three molecular detection methods (Friedman test, *p* < 0.001). The median estimated cell equivalents were 54.3 (IQR, 0–250.5), 17.7 (IQR, 0–40.7), and 0 (IQR, 0–17.7) per 25 g for dPCR, PMA-dPCR without enhancer, and PMA-dPCR with enhancer, respectively. Pairwise comparisons showed that PMA-dPCR yielded significantly lower estimated counts than dPCR without PMA (Wilcoxon signed-rank test with Bonferroni correction, *p* < 0.001). PMA-dPCR with enhancer further reduced the estimated counts compared with PMA-dPCR (*p* < 0.001). All quantitative results obtained in this study are summarized in Supplementary Table S2.

## 4 Discussion

In this study, we compared culture-based methods, dPCR without PMA, PMA-dPCR, and PMA-dPCR with enhancer for the detection of *Campylobacter* spp. in retail chicken meat collected over a 12-month period. Among the tested methods, dPCR without PMA showed the highest detection rate, whereas PMA treatment reduced positivity rates, and PMA-dPCR with enhancer showed the lowest detection rate. These findings suggest that a considerable proportion of signals detected by conventional nucleic acid amplification methods may originate from dead cells or bacterial populations with compromised membrane integrity.

Although PMA-dPCR without enhancer showed a numerically higher overall detection rate than either culture method, paired comparisons did not show statistically significant differences, indicating substantial discordance between the methods at the individual-sample level. Culture-based methods are widely used for *Campylobacter* detection, facilitating comparison with findings from previous surveillance studies. A recent culture-based study reported *Campylobacter* spp. contamination in 25.5% of retail poultry meat samples, further supporting the importance of retail poultry as a potential source of human exposure (Popa et al., 2025). *Campylobacter* spp. is highly sensitive to environmental stresses, including refrigeration, oxygen exposure, and nutrient limitation during poultry processing and storage (Yagi et al., 2022). Under these conditions, the organism may lose culturability but retain metabolic activity and potential infectivity by entering the VBNC state (Yagi et al., 2022). Therefore, culture-based methods alone may not fully capture membrane-intact *Campylobacter* populations that have lost culturability.

PMA treatment reduced the number of positive samples and detected copy numbers compared with dPCR without PMA. As mentioned earlier, PMA selectively penetrates membrane-damaged or dead cells and inhibits amplification of their DNA (Nocker et al., 2006; Hossain et al., 2025), suggesting that a portion of signals detected without PMA treatment likely originated from dead cells. It should also be noted that untreated dPCR samples did not undergo the additional incubation, light exposure, centrifugation, and washing steps required for PMA treatment. Therefore, differences between untreated dPCR and PMA-dPCR may partly reflect processing-related variation or loss of bacterial cells, in addition to the selective effect of PMA treatment. However, detectable signals remained in many samples after PMA treatment, indicating the presence of bacterial populations with relatively intact membrane structures.

Interestingly, the addition of PMA enhancer further reduced both positivity rates and copy numbers in several samples. According to the manufacturer’s instructions, PMA enhancer is designed to increase membrane permeability and improve exclusion of signals derived from membrane-compromised cells. In *Campylobacter* spp., this approach has previously been shown to reduce detection of injured or VBNC-associated populations (Hossain et al., 2025). Therefore, the difference between PMA-treated conditions with and without enhancer suggests that retail chicken meat may contain heterogeneous *Campylobacter* populations with varying physiological states. Some culture-negative, membrane-intact populations may include cells in the VBNC state, although this was not directly confirmed in the present study. These findings indicate that membrane integrity may vary across bacterial populations present in poultry meat products.

An apparent temporal pattern was observed, with higher detection rates during the summer months, particularly from July to September, and lower rates during early spring. This pattern was observed across both culture-based and molecular methods and is consistent with previous reports describing increased *Campylobacter* contamination during warmer seasons (Wallace et al., 1997; Meldrum et al., 2005). Environmental temperature, flock prevalence, and processing conditions may contribute to such temporal variation (Jorgensen et al., 2011; Sasaki et al., 2023).

This study has several limitations. First, only retail chicken meat samples from a limited geographical area were analyzed. Second, the VBNC state of the detected populations was not directly confirmed by physiological characterization, and interpretations were based primarily on membrane integrity-dependent molecular detection. Therefore, culture-negative, membrane-intact populations detected in this study should not be regarded as confirmed VBNC cells. Further studies using additional viability markers or resuscitation assays are needed to better characterize these bacterial populations. Third, bacterial cell equivalents were estimated assuming three copies of the 16S rRNA gene per cell based on previous reports for *C. jejuni* and *C. coli* (Taylor et al., 1992). Because the assay targets *Campylobacter* spp., potential variation in 16S rRNA gene copy number among species or strains may introduce uncertainty into these quantitative estimates. Fourth, formal limits of blank, detection, and quantification were not established in the present study. In our previous methodological study, increased variation among replicate dPCR measurements was observed at low target concentrations, including occasional non-detection in individual replicates (Hossain et al., 2025). Therefore, low-level positive results in the present study were interpreted as evidence of target detection but should not be regarded as robust quantitative measurements, and should be interpreted with consideration of both analytical and sampling variation. In addition, heterogeneous distribution of bacterial cells within the chicken-meat matrix may introduce sampling variation between separate aliquots, particularly at low bacterial concentrations. Therefore, low-level dPCR results should be interpreted with consideration of both analytical and sampling variation.

Despite these limitations, our findings demonstrate that PMA-dPCR provides a useful approach for evaluating membrane-intact *Campylobacter* populations in poultry meat. In particular, comparison of PMA treatment with and without enhancer may provide additional insight into differences in membrane integrity among *Campylobacter* populations present in retail chicken products. These approaches may contribute to the development of rapid and practical monitoring systems for poultry processing and food safety management.

## Data Availability

The original contributions presented in the study are included in the article/Supplementary material, further inquiries can be directed to the corresponding author.
